# Insights on Lipomatosis after Platinum-Based Chemotherapy Use in Pediatric Oncology: A Case Report

**DOI:** 10.3390/medicina58121715

**Published:** 2022-11-23

**Authors:** Estera Boeriu, Alexandra Georgiana Boc, Alexandra Borda, Rodica Anamaria Negrean, Bogdan Feciche, Amalia Iulia Boeriu, Florin George Horhat, Ion Cristian Mot, Ioana Delia Horhat, Madhavi Ravulapalli, Omar Sabuni, Abduljabar Adi, Adnan Anjary, Smaranda Teodora Arghirescu

**Affiliations:** 1 Department of Pediatrics, “Victor Babes” University of Medicine and Pharmacy, Eftimie Murgu Square 2, 300041 Timisoara, Romania; 2 Department of Oncology and Haematology, “Louis Turcanu” Emergency Clinical Hospital for Children, Iosif Nemoianu Street 2, 300011 Timisoara, Romania; 3 Faculty of Medicine and Pharmacy, University of Oradea, 410073 Oradea, Romania; 4 Department of Urology, Satu-Mare County Emergency Hospital, Strada Ravensburg 2, 440192 Satu-Mare, Romania; 5 Klinikum Landshut, Teaching Hospital of the LMU Munich, Clinic for Anaesthesiology, Intensive Care Medicine and Pain Therapy, Robert-Koch-Strasse, 184034 Landshut, Germany; 6 Multidisciplinary Research Center on Antimicrobial Resistance (MULTI-REZ), Microbiology Department, “Victor Babes” University of Medicine and Pharmacy, 300041 Timisoara, Romania; 7 Department of Ear-Nose-Throat, “Victor Babes” University of Medicine and Pharmacy Timisoara, Eftimie Murgu Square 2, 300041 Timisoara, Romania; 8 Bhaskar Medical College, Amdapur Road 156-162, Hyderabad 500075, India; 9 Faculty of General Medicine, Altinbas University, Dilmenler Cd., 34217 Istanbul, Turkey; 10 Faculty of General Medicine, Baskent University, Fatih Sultan, 06790 Ankara, Turkey; 11 Faculty of General Medicine, Yeditepe University, Kayısdagı Cd., 34755 Istanbul, Turkey

**Keywords:** pediatric oncology, lipomatosis, cisplatin, platinum-based chemotherapy, germ cell tumors

## Abstract

Agents of platinum-based chemotherapy, such as cisplatin or carboplatin, are used in the treatment of a wide range of malignancies that affect children, such as brain tumors, osteosarcoma, neuroblastoma, hepatoblastoma, and germ cell tumors (GCTs). The Cyclophosphamide Equivalent Dose (CED) calculator for reproductive risk does not take platinum-based chemotherapy into account, despite the fact that it accounts for the majority of chemotherapy medications that are typically administered for pediatric GCTs. As a result, exposure to platinum-based drugs throughout infancy can have predictable long-term effects such as infertility, as well as other rare encounters such as lipoma formation and lipomatosis. Lipomas are the most prevalent benign soft tissue tumor subtype. They may be either solitary entities or engaged in multiple lipomatosis, which may have a familial origin or be an acquired disorder. Chemotherapy is a possible cause of lipomatosis. Chemotherapy based on cisplatin has been linked to a variety of long-term consequences, including kidney damage, neurotoxicity, and pulmonary toxicity, and may even create secondary cancers. However, lipoma development is known to occur in fewer than 1 in 100 individuals, and only a few examples of multiple cutaneous lipomatosis triggered by this therapy have been documented. Here we present a very rare case of lipomatosis in a pediatric patient with GCT under cisplatin therapy, which might be the third report of this kind affecting children.

## 1. Introduction

Lipomas are benign, most often encapsulated, noninvasive soft tissue tumors composed of mature adipocytes. They represent about half of all the adipocytic tumors in the pediatric population and, depending on their size and location, and they vary from being asymptomatic to causing various non-specific symptoms. Cytogenetic aberrations can often be described in these tumors, most of them affecting the chromosome 12q. The term “lipomatosis”, on the other hand, describes tumors comprised of matured adipose tissue with an infiltrative growth pattern, ill-defined margins, and a tendency toward symmetry [1,2,3].

While the cause of these tumors remains mostly unknown, there are some etiologically and clinically described subtypes of lipomatosis, such as Madelung’s disease associated with alcohol consumption and metabolic problems; lipomatosis dolorosa, potentially associated with improper lymph drainage; or Dercum’s disease and Roch–Leri mesosomatous lipomatosis, which are considered very rare lipomatoses of unknown etiology [4,5]. There have been cases reported where it seems that the medical intervention, whether through the administered medication or surgery, might have either caused or exacerbated the growth of lipomatous tumors [6,7,8]. Among the incriminated drugs lies cisplatin, a platinum-based compound with a cytotoxic effect used in the treatment of various cancers [9,10,11,12]. Due to its side effects and drug resistance, cisplatin is currently used in combination with other antineoplastic agents. Through oxidative stress, high doses of this drug can induce nephrotoxicity and hepatotoxicity, demonstrated by elevated creatinine levels, hepatic enzyme levels, and bilirubin levels. Necrosis and inflammatory cell infiltration of the portal area are some of the histopathological changes observed in patients treated with cisplatin [13]. Other platinum-based agents have been demonstrated to induce hepatosteatosis, a condition that has been linked to the growth of lipomas [14,15].

In this case report, we present a very rare case of a 16-year-old girl diagnosed with a giant ovarian GCT of mixed histology with extension into the abdominal cavity and asymptomatic amputation of the contra-lateral ovary, who developed lipomatosis after chemotherapy with cisplatin. A thorough literature search was also performed to find similar cases, since this rare occurrence of peritoneal and liver lipomas as a possible and specific side effect of platinum-based chemotherapy is scarcely described in pediatric patients.

## 2. Case Presentation

A 16-year-old Caucasian girl was admitted to our hospital, presenting with abdominal pain and abdominal distension, fever, and a dry cough. Upon clinical examination, the patient was found moderately pale and febrile, with a body mass index (BMI) of 28.6 kg/m^2^, being in the 95th percentile for girls her age, corresponding to class I obesity. The abdomen was distended and moderately painful upon palpation, the puddle sign was present, and percussion notes varied between tympanic and dull, raising suspicion of ascites. Lab values were significantly modified, suggesting a severe inflammatory process, as described in Table 1.

It was observed that CRP levels were 3215 mg/l, ferritin 1118 ng/mL, fibrinogen 629 mg/dL, and ESR was 92 mm/h, all of which ranged much higher than the normal threshold. Besides the severe inflammatory status of the patient, the girl was also suffering from anemia, with a red blood cell count (RBC) measured at 4.0 million, and a hemoglobin level of 10.5 g/dL. Apart from class I obesity, the adolescent patient had an elevated serum glucose (109 mg/dL), correlating with a metabolic syndrome. The serum lipid levels were also above the normal range for adolescents, although without a significant increase (total cholesterol = 183 mg/dL, triglycerides = 155 mg/dL). The patient did not have any documented endocrinological problems.

A chest X-ray found bilateral interstitial reticular coarseness and opacification of the right costophrenic angle, suggestive of pleural effusion. The patient was further investigated in our ultrasound department; the abdominal sonogram raised suspicion of an abdominal–pelvic tumor mass with mild ascites in the abdominal cavity, particularly pronounced in the perihepatic and peri-splenic spaces.

**Figure 1:**
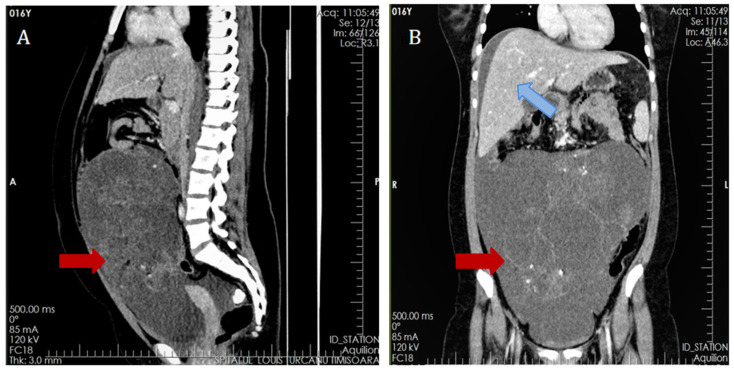
CT scan revealed a massive heterogeneous tumor mass of 18/13/9 (red arrow) cm with a volume of 1101 mm^3^, of pelvic origin with extension in the abdominal cavity manifesting compression on the local organs. Perihepatic ascites can be easily identified (blue arrow).

Surgery was considered and computer tomography (CT) was performed on the same day to better assess the extension of the tumor mass. The CT examination of the abdominal and pelvic cavities found a gigantic heterogeneous mass of 18/13/9 cm (Figure 1). The tumor did not invade the abdominal organs or retroperitoneal vascular structures, but manifested a compressive effect on the intestinal loops and urinary bladder. While the right ovary could not be visualized, a calcified mass of 26/14/12 mm was identified within the right anterolateral side of the recto-uterine pouch.

The major findings could be summarized as massive mixed solid-cystic tumors occupying the abdominal and pelvic cavities of suspected ovarian origin, abdominopelvic ascites, minimal right pleural effusion, and para-aortic lymphadenopathy. The patient underwent surgery on the third day of hospitalization. A midline laparotomy found peritoneal fluid that was drained and sampled for cytology and a massive septated cystic tumor occupying the abdominal and pelvic cavity with left ovarian origin. A surgical biopsy was performed along with ablation of the ovarian tumor and epiploectomy. Upon further inspection of the pelvic cavity, a calcified amputated right ovary of approximately 3cm in diameter was found, most likely a sequela of compression due to the massive volume of 1101.43mm^3^ from the tumor originating in the contralateral ovary. The patient was transferred to our intensive care unit for post-surgery monitoring awaiting the pathology and cytology results.

The cytologic examination of the peritoneal fluid identified hemorrhagic fluid with high heterogeneous cellularity of 75% neutrophils, 15% monocytes, 10% lymphocytes, reactive mesothelial cells, and several old mesothelial cells with “signet ring cell” phenotype, raising suspicion of peritoneal dissemination. Pathology findings revealed on macroscopic examination the tumor appeared as a round, encapsulated multi-cystic mass of 18.5/13.8/9.5 cm, with grey to light pink solid/gelatinous components. Immunohistochemistry (IHC) analysis found a glial fibrillary acidic protein (GFAP), alpha-fetoprotein, CD10, macro creatine kinase (MCK), and vimentin, all intensely positive, beta-HCG-negative, and KI-67-positive in 10% of solid tumor areas [16,17,18].

**Figure 2:**
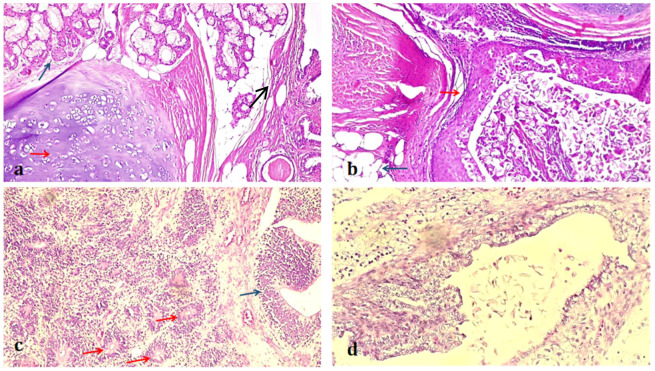
Histological examination revealed a tumor of germ cell origin composed of areas of mature teratoma, immature teratoma, and small areas of yolk sac tumor. (**a**). Teratoma with non-specific glandular tissue (blue arrow) and cartilaginous tissue (red arrow). (**b**). Teratoma shows areas of the dispersed squamous-stratified epithelium (red arrow), adipocytes (blue arrow), and immature smooth muscle cells (black arrow). (**c**). The major component of the tumor consisted of immature teratoma histology, with immature neuroectodermal tissue forming rosettes (red arrows) and tubules (blue arrows). (**d**). Detail of yolk sac component with glandular growth pattern.

The pathology findings identified the tumor as a mixed germ cell tumor with three components, as observed in Figure 2: mature teratoma (25%), immature teratoma, high-grade G3 (60%), and yolk sack tumor (15%). The tumor was graded at stage IV (pT1cNxM1) due to evidence of metastasis, according to the TNM staging system [19], where “p” and “c” stand for staging based on the histopathological assessment of a surgical specimen and evidence acquired by clinical and paraclinical procedures, respectively.

A second pathology examination was requested, and the result indicated a high-grade immature teratoma. The final diagnosis was set after careful consideration of clinical, paraclinical, histopathological, and IHC data. The tumor was labeled stage IIIC according to the FIGO staging system that identified peritoneal metastasis [20], and a high-grade/G3 immature teratoma of the ovary [1].

Therapy and follow-up: the patient underwent chemotherapy treatment (MAKEI 2005 protocol) with four PEI cycles (cisplatin, etoposide, and ifosfamide). The chemotherapy was well tolerated with no significant events, and complete tumor remission was accomplished after the completion of the four PEI cycles. Shortly after completing the last cycle of chemotherapy, a routine MRI showed elements previously absent four months prior: a small oval peripheral intrahepatic mass of ~0.8 cm under the Glisson capsule and several smaller cysts in the perihepatic space. A PET scan was further performed and found no signs of malignancy but identified the liver mass as a possible lipoma. The tumor markers AFP and beta-HCG were within normal values; thus, a “watch and wait” approach was decided upon.

The routine check-ups involving physical examination, lab values, tumor markers, and imaging studies showed no signs of relapse or significant changes in the patient’s health, apart from mild hepatic steatosis and significant weight gain (IMC = 35.4 kg/m^2^), advancing the previously diagnosed obesity to class II. No endocrine causes for the patient’s weight gain were found. The iatrogenic menopause following bilateral adnexectomy was well controlled by hormone substitution therapy, therefore the weight gain was attributed to genetics, an admittedly unbalanced diet, and a sedentary lifestyle.

**Figure 3:**
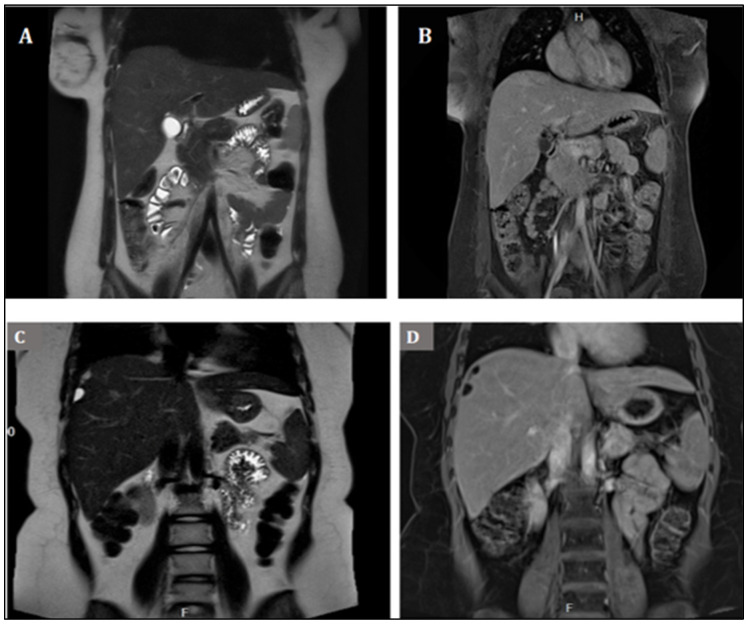
Coronal MRI of the abdominal cavities. (**A**). MRI T2-weighted performed after the surgical procedure, immediately before the start of chemotherapy. (**B**). Contrast-enhanced T1-weighted sequence in the same examination. (**C**). MRI T2-weighted of the abdominal cavity 18 months after completion of chemotherapy shows hyperintense perihepatic and subdiaphragmatic masses. (**D**). T1-weighted fat-suppressed (Dixon) sequence revealed the masses to display low intensity, identifying the small tumors as fat structures rather than liquid containing possible lipomas, an assumption later confirmed after surgical excision.

In the 18th month since the end of chemotherapy, a routine MRI found that the perihepatic masses had increased in number and dimension. The investigation found intrahepatic, perihepatic, and subdiaphragmatic fat-containing lesions of a maximum of 2/1.5 cm (Figure 3C,D), a smaller mass of approximately 1.1/0.6 cm in the hepatorenal recess, and another of 0.4 cm in the gastrosplenic recess. Tumor markers AFP, b-HCG, and CA-125 were normal; therefore, the rare growing teratoma syndrome (GTS) was feared as a severe outcome [21,22]. Five tumor masses were removed by exploratory laparotomy two weeks later, and the pathology exam found all the lesions to be lipomas.

## 3. Discussion

### 3.1. Literature Search for Similar Findings

We report a case of a female pediatric patient that presented with systemic lipomatosis after cisplatin therapy for GCT and no familial history of lipomatosis. Lipomatosis is not an uncommon side effect of chemotherapy, and it is generally linked to the use of corticosteroids [23]. Since our patient did not undergo steroid therapy and considering the alarmingly rapid growth of the number of lipomas, we investigated the possibility that her chemotherapy regimen might be the culprit. A quick scan of the literature revealed no reports of lipomatosis involving exposure to etoposide or ifosfamide, but a likely link to cisplatin, and a reported side effect of lipoma formation is fewer than 1 in 100 cases.

**Figure 4:**
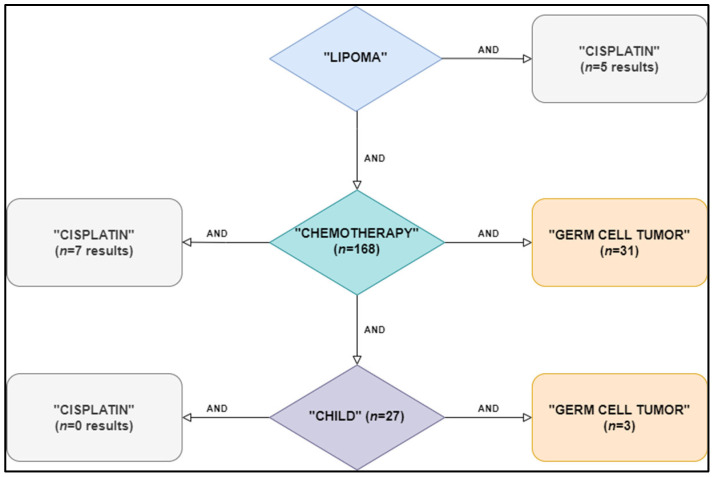
Flowchart of steps and keywords used for database search.

An extensive literature search using the terms “lipomatosis”, “lipoma”, “chemotherapy”, “cisplatin” or “carboplatin”, “platinum-based chemotherapy”, and “germ cell tumor” was conducted using the “PubMed”, “Web of Science”, and “Scopus” databases in an attempt to answer the following question: how often are singular or multiple lipomas associated with the use of cisplatin or other platinum agents? Articles in the English literature published between 2000 to 2021 were included in our final search. The main steps of our literature scan are presented in Figure 4. We excluded articles irrelevant to our query and reports of cases where underlying genetic or metabolic conditions associated with a high incidence of lipomatous tumors were present (e.g., Cowden syndrome, Cushing’s syndrome, Rb1 mutation, familial multiple lipomatosis, hamartoma tumor syndrome). A history of corticosteroid therapy was also an exclusion criterion due to its well-known association with the development of lipomatosis [23].

The only two cases found relevant to our search described intestinal and cutaneous lipomatosis occurring in two adult cancer survivors who underwent cisplatin therapy [24,25]. Cutaneous lipomatosis was described as the most prevalent benign mesenchymal tumor, without a significant comorbid problem, that forms in the subcutaneous tissues of the trunk or proximal extremities and manifests as distinct rubbery lumps. Although in one report the lipomas were discovered 12 years after cisplatin therapy, it is a known fact that circulating platinum levels remain high decades after the end of the regimen [26]. The first reported patient was diagnosed with testicular non-seminoma GCT treated with a right orchiectomy and a treatment scheme using bleomycin, etoposide, and cisplatin, followed by bilateral aortoiliac embolectomies during BEP treatment. The role of chemotherapy as a cause or aggravator of intestinal lipomatosis has not been conclusively proven [27,28]. Notably, the development and expansion of the lipomas seen in this patient’s follow-up studies after chemotherapy treatment imply a causal involvement of the chemotherapy, but the potential of a congenital illness with a late presenting phenotype and limited penetrance persists. Multiple case reports demonstrate chemotherapy-induced cutaneous lipomatosis, although many of these patients had previously received steroids, which have a well-established relationship with acquired lipomatosis.

Significantly, the second case report described a patient with testicular cancer who was also treated with BEP chemotherapy and acquired cutaneous lipomatosis; it was hypothesized that chemotherapy might cause adipocyte proliferation via a mechanism that has yet to be determined [25]. Nonetheless, the patient had received a peripheral blood stem cell transplant, but there are no previous reports of multiple lipomatosis developing after such procedures. Regarding stem cell mobilization and transplant, there are no published reports of adipose cell proliferation as a well-known adverse effect. However, it would be beneficial to be able to show that chemo-mobilization agents may also drive adipocyte proliferation in addition to blood cells [29]. In fact, the stimulating effect of the Macrophage Colony-Stimulating Factor (MCSF) on adipocytes is well-established, although little is known about GCSF. In spite of this, recent research indicates a link between the increased release of GCSF and the expansion of cultured adipocyte sizes [30].

### 3.2. Germ Cell Tumors in Pediatric Oncology

The presented case represents a very rare occurrence in pediatric oncology, both by the mixed histology of the GCT and even more for the association of platinum-based chemotherapy with the development of systemic lipomatosis. Regarding the malignant diagnosis in this patient, it is important to acknowledge the rarity of a mixed GCT in pediatric oncology [31,32]. Germ cell tumors are a group of rare and highly heterogeneous neoplasms with respect to their site of presentation, histology, age of onset, and molecular signature [33,34]. Their incidence follows a bimodal curve, particularly high in children under four years of age and another peak later in adolescence. The highest incidence of GCT is seen in infants up to one month, where they account for 30–45% of neonatal tumors, although only 5% are proven malignant [33]. While GCT represents 3.6% of all cancers before puberty, their incidence spikes up to 12% in the 15–19-year age group that defined our presented case. In Europe, as well as in the USA and Asia, the incidence is significantly higher in males versus females, with 64 cases per million for males and 4 per million in female patients, such as our finding [35].

In pediatric patients, a quarter of all malignant GCT presents two or more histological types, thus classified as mixed germ cell tumors [36,37]. In females, the ovary is the most common site for GCT, with an incidence of 1 in 400 thousand in Europe, thus it is a rare cancer by RARECARE definition with an incidence that falls below 6 in 100 thousand cases [35]. GCTs make up 25% of all diagnosed ovarian masses and 3-5% of malignant ovarian cancers, as presented in this particular case. They are the most frequent ovarian malignancies in women under 25 years of age [38,39]. Although mixed GCT_S_ of the ovary are exceptionally rare (less than 1% of ovarian GCTs), they are the most common non-seminomatous tumors in adolescent girls, having the lowest 5-year relative survival rate in the 10–19 age group [37,40].

As presented in our rare case, surgical intervention was fundamental in the diagnosis and management of the large tumor, and therefore it is often the first step of the treatment in the majority of large-volume tumors. A laparotomy is usually performed, given the size and solid nature of these tumors. Since germ cell tumors occur mostly in the younger population, when possible, fertility-sparing interventions are performed in which the unaffected ovary and fallopian tube are preserved [41,42,43,44]. In cases of locally extended tumors or recurrent tumors, pre-surgical chemotherapy is preferred in order to reduce the tumor size and ensure better chances of a second step, complete carcinogenic surgical resection [45]. For GCTs, chemotherapy often involves the use of etoposide, ifosfamide, or cisplatin, a platinum-based drug that was used for the 16-year-old girl presented in our case [46]. However, when a clear diagnosis cannot be made before surgery, or the volume of the tumor creates a life-threatening circumstance, surgery will be performed before the chemotherapy management.

### 3.3. Platinum-Based Chemotherapy in Pediatric-Oncology

While in adults any immature teratoma classified higher than stage 1, based on the FIGO system, and a lower differentiation than Grade 1A, would be treated with chemotherapy without considering prior surgical management, in pediatric patients the use of chemotherapy has been challenged, with authors suggesting that complete resection of the tumor alone is sufficient, sometimes regardless of the grading, with no significant differences in overall 4–5 year survival of these patients compared to those who receive platinum-based chemotherapy [26,47,48,49]. It is worth noting, however, that literature on refractory and relapsed germ cell tumors in the pediatric population is scarce. Tumors that have previously been treated by surgery alone continue to have an excellent prognosis; however, in cases formerly treated with antineoplastic drugs, survival rates drop quite significantly. The main prognostic factor in such situations seems to come down to whether or not complete resection is possible. In order to achieve this, platinum-based chemotherapy is recommended beforehand [45].

Although chemotherapy treatment guidelines are clear in adult patients, they become uncertain in the pediatric population when involving ovarian GCTs of mixed histology due to the very low incidence of these cases. The use of cisplatin-based chemotherapy has been a breakthrough, not only in the treatment of gynecological cancers, but also in neuroblastomas, hepatoblastomas, medulloblastomas, and other solid tumors, drastically improving survival rates, and several studies have proven its superiority over carboplatin. This observation has led to the introduction of cisplatin in most chemotherapy schemes (the exception being the British protocol, which uses carboplatin) in combination with etoposide, ifosfamide, bleomycin, or vinblastin [50]. The downside of using platinum-based drugs is that blood serum platinum concentration remains high nearly 20 years after receiving cytotoxic therapy [51]. Platinum agents and long-term exposure to circulating platinum have been associated with long-term sequelae such as paresthesia, lipid disorder, hypertension, and gonadal dysfunction, as well as an increased risk of second malignancies and delayed cardiotoxicity [52].

The downside of using platinum-based chemotherapy is the risk of developing secondary tumors. This theory was substantiated by research that discovered a dose-dependent impact of chemotherapy on GCTs in 96 chemotherapy-treated individuals. In larger cohort studies, a dose-dependent link between platinum-based chemotherapy and GCTs eradication has not been studied [53]. In addition, in contrast to our case involving a 16-year old female patient with an ovarian GCT, the documented occurrences of contralateral tumoral recurrence were exclusively in male individuals. Moreover, all patients were young adults, with no reports being available on the recurrence of GCT due to platinum-based chemotherapy. Whether or not such a correlation exists is clinically significant since a growing number of patients with testicular GCTs may receive lower doses of platinum-based chemotherapy as an adjuvant treatment with one or two rounds of chemotherapy for high-risk early-stage illness gains popularity.

### 3.4. Platinum-Based Chemotherapy and Lipomatosis

Even though the underlying process is uncertain, cisplatin-based treatment might be a causative factor, although without significant proof. Cisplatin has an alkylating-like property, binding DNA and inducing apoptosis. Even if the growth of fat cells created by an antimitotic agent may seem counterintuitive, it is vital to recognize the alterations in the body caused by cisplatin. In reality, an increased body mass index and metabolic syndrome are potential side effects that may be attributed to hormonal changes brought on by chemotherapy, such as a testosterone deficit.

Quite the contrary, it has been demonstrated that cisplatin inhibits the migration and differentiation potential of human adipose-derived mesenchymal stem cells, and it can be assumed that the same mechanism is applicable to peripheral blood stem cells [54]. Adipose stem cells show promise in the repair of cytotoxic tissue injury in vitro and in vivo animal studies [55,56]. This is due to their chemotropism for sites of cell injury and inflammation, as well as their potential to differentiate into various cell lines under the right stimuli [57]. Chang et al. showed that adipose stem cells from subcutaneous adipose tissue collected from a patient who underwent cisplatin therapy four months prior displayed a significantly reduced differentiation capacity compared to those sampled from a healthy donor.

Our patient showed no signs of preexisting lipomas or abnormal growth in the affected area on the MRI performed before and after surgery prior to the start of platinum-based chemotherapy. The first signs of the abnormal growth could be seen less than a month after the last platinum agent-based cycle (cisplatin), the slight increase in size (~1–2 mm) was confirmed six months later, and surgery became necessary 18 months after the last PEI cycle (Figure 3C,D). Although the time correlation of cisplatin therapy with the onset of lipomatosis in our patient seems to be strong, it cannot be excluded that other factors might have played a significant role in this condition. Obesity may have been associated with an increased release of adipocyte-produced steroidogenic enzymes, leading to increased paracrine estrogen release [58]. Moreover, estrogen concentrations during hormone replacement therapy after bilateral adnexectomy may have differed from those occurring physiologically. In light of this and given the fact that only two cases of lipomatosis after CDDP therapy have been reported, the effect of estrogens seems more significant.

While the occurrence of lipomas after non-steroid chemotherapy regiments has not been frequently reported, chemotherapy-associated fatty depositions and liver injury is a well-known phenomenon that can manifest itself in a wide range of patterns from transient transaminitis, cholestasis, and venous occlusion to acute liver failure or neoplastic transformation. Cisplatin, one of the antineoplastic agents that our patient has received, has been noted to temporarily elevate liver enzymes, but it is rarely linked to severe cases of hepatotoxicity and fatty infiltration of the liver. However, another platinum-based agent, namely oxaliplatin, has been recently associated with the development of hepatosteatosis in adults who underwent hepatic resections due to colorectal or hepatic metastases [59]. One study that aimed to evaluate whether such lesions were evident in the pediatric population as well failed to find severe histopathological modifications, such as steatosis, fibrosis, or necrosis [15]. The reason why such findings appear in adult patients and not pediatric patients remains unknown, but it could be explained by the different drug combinations used in the pediatric population compared to the adult population, as well as by the shorter period of time elapsed between the end of neoadjuvant chemotherapy cycle and surgery in pediatric patients.

In the case of our patient, it would be unwise to disregard the nutritional factor that might have played a role in the development of the fatty liver. In the midst of the obesity epidemic, which has unfortunately not spared children, non-alcoholic fatty liver disease (NAFLD) has become the number one cause of chronic hepatic disorders in both children and adults [60]. Moreover, a statistically significant relationship between liver steatosis and hepatic lipomas was found with 50% of them also correlating with steatosis, and two-thirds of the lipomas were associated with obesity and hypercholesterolemia [61]. However, our patient had an increased BMI prior to the surgery and chemotherapy; therefore, obesity alone might not necessarily be the only factor involved in the development of lipomas.

Another factor that should be taken into account when discussing the etiology of the lipomatosis that our patient developed is the hormonal replacement therapy (HRT) administered after surgery. Although the process is not yet known, estrogen seems to promote the subcutaneous accumulation of adipose tissue by inhibiting lipolysis in the subcutaneous deposits only. That way, intra-abdominal fat deposits, which are typically seen in the male population and post-menopausal women, are shifted predominantly toward the subcutaneous regions. After menopause, either physiological or medically induced, a redistribution of fat is observed, with fat starting to accumulate in the visceral area. HRT, among other treatments, prevents both visceral fat distribution and additional weight gain [62,63]. Given that the HRT received by our patient was carefully monitored and deemed adequate, we consider it unlikely to have played a role in the development of lipomatosis.

The two phenomena described as “Chemotherapeutic retroconversion” and “growing teratoma syndrome” could explain the lipomatosis that appeared in our patient after chemotherapy [64,65]. Mature teratomas are germ cell tumors composed of different cell types, with the potential to mimic any tissue type of the body. According to the previously mentioned concepts, chemotherapy either contributes to the maturation of the immature cells in the immature teratomas or induces apoptosis in the immature cells, allowing the mature and slow-dividing cells to flourish. These theories were tested in a Japanese descriptive study published in 2021 [65] by analyzing the response of embryonic stem cells and induced pluripotent stem cell-derived immature teratomas to cisplatin in mice. The study showed that, after being treated with cisplatin, the tumors increased their maturation score, and on histological examination, mature tissues were found. Also, after treatment with cisplatin, the tumors exhibited immunostaining a lower immaturity index. Thus, the study concluded that cisplatin not only preferably destroys immature cells but also induces maturation in the teratoma tissue.

## 4. Conclusions

Despite the existing evidence, lack of genetic analysis of the patients and experimental methods to determine causality leave the possible association between the use of platinum-based agents and the development of lipomatosis uncertain. The underlying mechanism by which cisplatin or other platinum agents might affect adipocytes and favor the development of lipomatous tumors is yet to be understood. This case report and literature review provides further important evidence into a possible very rare side effect of cisplatin, although it requires further research and in vitro studies. Until more data is available, we suggest that our fellow clinicians consider previous treatment with platinum agents as a possible cause in cases of rapid development and growth of lipomas when nutritional, hormonal, or familial predisposition do not warrant the clinical manifestations.

## Figures and Tables

**Table 1:** Modified laboratory values significant for diagnosis.

Test	Value	Range	Significance
RBC (female)	4.0 × 10^9^/mm^3^	4.2–5.4 × 10^9^/mm^3^	↓
Hemoglobin	10.5 g/dL	11.8–15.7 g/dL	↓
Leukocytes	17.200/mm3	4.800–10.800/mm^3^	↑
Neutrophils	13.810/mm3	1870–8100/mm^3^	↑
Serum glucose	109 mg/dL	70–100 mg/dL	↑
Total cholesterol	183 mg/dL	<170 mg/dL	↑
Triglycerides	155 mg/dL	<150 mg/dL	↑
Serum albumin	4.1 mg/dL	3.4–5.4 g/dL	Within normal range
CRP	3215 mg/L	0–5 mg/L	↑
Ferritin	1118 ng/mL	15–150 ng/mL	↑
Fibrinogen	629 mg/dL	200–400 mg/dL	↑
Procalcitonin	0.244 ng/mL	0–0.5 ng/mL	Within normal range
ESR	92 mm/h	2–13 mm/h	↑
AFP	–	–	–
β-HCG	–	–	–
CA-125	–	–	–

Tumor markers were not determined prior to surgery; CRP—C-reactive protein; ESR-Erythrocyte sedimentation rate; AFP—Alpha-fetoprotein; β-HCG—beta human chorionic gonadotropin; CA-125—Cancer antigen 125. ↓: below normal range; ↑: above normal range.

## References

[1] Hashizume N., Aiko T., Fukahori S., Ishii S., Saikusa N., Koga Y., Higashidate N., Sakamoto S., Tsuruhisa S., Nakahara H. (2020). Benign mesenteric lipomatous tumor in a child: A case report and literature review. Surg. Case Rep..

[2] Putra J., Al-Ibraheemi A. (2019). Adipocytic tumors in Children: A contemporary review. Semin. Diagn. Pathol..

[3] Schiltz D., Mueller K., Ortner C., Tschernitz S., Anker A., Felthaus O., Schreml J., Koller M., Prantl L., Schreml S. (2021). Multiple Symmetric Lipomatosis: A Cross-Sectional Study to Investigate Clinical Features and Patients’ Quality of Life. Symmetry.

[4] Al Ghazal P., Grönemeyer L.-L., Schön M.P. (2018). Lipomatosen. JDDG J. Dtsch. Dermatol. Ges..

[5] Lemaitre M., Aubert S., Chevalier B., Jannin A., Bourry J., Prévost G., Lefebvre H., Vantyghem M.-C. (2021). Rare Forms of Lipomatosis: Dercum’s Disease and Roch-Leri Mesosomatous Lipomatosis. J. Clin. Med..

[6] Sollier C., Capel E., Aguilhon C., Smirnov V., Auclair M., Douillard C., Ladsous M., Defoort-Dhellemmes S., Gorwood J., Braud L. (2021). LIPE-related lipodystrophic syndrome: Clinical features and disease modeling using adipose stem cells. Eur. J. Endocrinol..

[7] Gudjoncik A., De Carvalho A., Loris L., Chavent A., Cercueil J.-P., Jazayeri S., Cottin Y. (2008). Postoperative mediastinal lipomatosis. Arch. Cardiovasc. Dis..

[8] Humblot S., Weber J., Korganow A.-S., Hammann B., Pasquali J., Martin T. (1997). Les lipomatoses induites par la corticothérapie. Rev. Méd. Interne.

[9] Dasari S., Tchounwou P.B. (2014). Cisplatin in cancer therapy: Molecular mechanisms of action. Eur. J. Pharmacol..

[10] Sharmin S., Rahaman M., Martorell M., Sastre-Serra J., Sharifi-Rad J., Butnariu M., Bagiu I.C., Bagiu R.V., Islam M.T. (2021). Cytotoxicity of synthetic derivatives against breast cancer and multi-drug resistant breast cancer cell lines: A literature-based perspective study. Cancer Cell Int..

[11] Brown A., Kumar S., Tchounwou P.B. (2019). Cisplatin-Based Chemotherapy of Human Cancers. J. Cancer Sci. Ther..

[12] Iacob R., Manolescu D.L., Stoicescu E.R., Fabian A., Malita D., Oancea C. (2022). Breast Cancer—How Can Imaging Help?. Healthcare.

[13] Ranasinghe R., Mathai M.L., Zulli A. (2022). Cisplatin for cancer therapy and overcoming chemoresistance. Heliyon.

[14] Tsuji K., Doyama H. (2017). S-1 induced hepatic steatosis in patients with pancreatic cancer: Retrospective analysis. World J. Gastrointest. Oncol..

[15] Martin-Benitez G., Marti-Bonmati L., Barber C., Vila R. (2012). Hepatic lipomas and steatosis: An association beyond chance. Eur. J. Radiol..

[16] Kucharz E.J., Kopeć-Mędrek M., Kramza J., Chrzanowska M., Kotyla P. (2019). Dercum’s disease (adiposis dolorosa): A review of clinical presentation and management. Reumatologia.

[17] Folescu R., Levai C.M., Grigoraş M.L., Arghirescu T.S., Talpoş I.C., Gîndac C.M., Zamfir C., Poroch V., Anghel M.D. (2018). Expression and significance of Ki-67 in lung cancer. Rom. J. Morphol. Embryol..

[18] Uxa S., Castillo-Binder P., Kohler R., Stangner K., Müller G.A., Engeland K. (2021). Ki-67 gene expression. Cell Death Differ..

[19] Grigoraş M.L., Arghirescu T.S., Folescu R., Talpoş I.C., Gîndac C.M., Zamfir C., Cornianu M., Anghel M.D., Levai C.M. (2017). Expression of E-cadherin in lung carcinoma, other than those with small cells (NSCLC). Rom. J. Morphol. Embryol..

[20] Rosen R.D., Sapra A. (2022). TNM Classification. StatPearls [Internet].

[21] Benedet J.L., Bender H., Jones H., Ngan H.Y., Pecorelli S. (2000). FIGO staging classifications and clinical practice guidelines in the management of gynecologic cancers. FIGO Committee on Gynecologic Oncology. Int. J. Gynaecol. Obstet..

[22] Rebelo J., Moreira F., Morgado M., Preto A.S., Madureira A. (2021). Growing Teratoma Syndrome: A Rare Outcome. Case Rep. Urol..

[23] Gurguş D., Grigoraş M.L., Motoc A.G.M., Zamfir C.L., Cornianu M., Faur C.I., Pop D.L., Folescu R. (2019). Clinical relevance and accuracy of p63 and TTF-1 for better approach of small cell lung carcinoma versus poorly differentiated nonkeratinizing squamous cell carcinoma. Rom. J. Morphol. Embryol..

[24] Hu S., Mojtahed A., Covington A., Thompson W., Volpicelli N., McCarthy D. (2016). Intestinal Lipomatosis and Chemotherapy: A Growing Concern. Am. J. Dig. Dis..

[25] Bracaglia R., D’Ettorre M., Gentileschi S., Mingrone G., Tambasco D. (2014). Multiple lipomatosis after stem cell trasplant and chemotherapy: A case report. Eur. Rev. Med. Pharmacol. Sci..

[26] Gietema J.A., Meinardi M.T., Messerschmidt J., Gelevert T., Alt F., Uges D.R., Sleijfer D.T. (2000). Circulating plasma platinum more than 10 years after cisplatin treatment for testicular cancer. Lancet.

[27] Cronin P.A., Myers E., Redmond H.P., O’Reilly S., Kirwan W.O. (2010). Lipomatosis: An unusual side-effect of cytotoxic chemotherapy?. Acta Derm. Venereol..

[28] Gotoh M., Kitahara T., Sakuta J., Akahane D., Ohyashiki K. (2010). Multiple lipoma with hyperlipidemia in a multiple myeloma patient treated with bortezomib/dexamethazone. Leuk. Res..

[29] Levine J., Jensen M., Eberhardt N., O’Brien T. (1998). Adipocyte macrophage colony-stimulating factor is a mediator of adipose tissue growth. J. Clin. Investig..

[30] Skurk T., Alberti-Huber C., Herder C., Hauner H. (2007). Relationship between Adipocyte Size and Adipokine Expression and Secretion. J. Clin. Endocrinol. Metab..

[31] Jawass M.A., Alezzi J., Bin Gouth H.S., Bahwal S.A., Bamatraf F.F., Ba’Amer A.A. (2016). Pattern of malignancies in children <15 years of age reported in Hadhramout Cancer Registry, Yemen between 2002 and 2014. Saudi. Med. J..

[32] Jinca C., Petrescu C.A.M., Boeriu E., Oprisoni A., Balint-Gib L., Baica M., Popa C., Andreescu N., Serban M., Ursu E. (2018). The impact of immunological and biomolecular investigations on the outcome of children with acute lymphoblastic leukemia—Experience of IIIrd Paediatric Clinic Timisoara. Rev. Romana Med. Lab..

[33] Mosbech C.H., Rechnitzer C., Brok J.S., Rajpert-De Meyts E., Hoei-Hansen C.E. (2017). Recent Advances in Understanding the Etiology and Pathogenesis of Pediatric Germ Cell Tumors. J. Pediatr. Hematol. Oncol..

[34] Trama A., Berrino F., Nogales F.F., Jimenez R.E. (2017). The Epidemiology of Malignant Germ Cell Tumors: The EUROCARE Study. Pathology and Biology of Human Germ Cell Tumors.

[35] Steliarova-Foucher E., Colombet M., Ries L.A.G., Moreno F., Dolya A., Bray F., Hesseling P., Shin H.Y., Stiller C.A., IICC-3 contributors (2017). International incidence of childhood cancer, 2001–2010: A population-based registry study. Lancet Oncol..

[36] Murray M.J., Nicholson J.C., Coleman N. (2015). Biology of childhood germ cell tumours, focussing on the significance of microRNAs. Andrology.

[37] Zambrano E., De Stefano D.V., Reyes-Múgica M., Nogales F.F., Jimenez R.E. (2017). Pediatric Germ Cell Tumors. Pathology and Biology of Human Germ Cell Tumors.

[38] Ulbright T.M. (2005). Germ cell tumors of the gonads: A selective review emphasizing problems in differential diagnosis, newly appreciated, and controversial issues. Mod. Pathol..

[39] Jimenez R.E., Gupta S., Herrera-Hernandez L.P., Sebo T.J., Nogales F.F., Jimenez R.E. (2017). Testicular Germ Cell Tumors. Pathology and Biology of Human Germ Cell Tumors.

[40] Smith H.O., Berwick M., Verschraegen C.F., Wiggins C., Lansing L., Muller C.Y., Qualls C.R. (2006). Incidence and Survival Rates for Female Malignant Germ Cell Tumors. Obstet. Gynecol..

[41] You W., Dainty L.A., Rose G.S., Krivak T., McHale M.T., Olsen C.H., Elkas J.C. (2005). Gynecologic Malignancies in Women Aged Less Than 25 Years. Obstet. Gynecol..

[42] Fonseca A., Frazier A.L., Shaikh F. (2019). Germ Cell Tumors in Adolescents and Young Adults. J. Oncol. Pract..

[43] Triarico S., Capozza M.A., Mastrangelo S., Attinà G., Maurizi P., Ruggiero A. (2020). Gynecological cancer among adolescents and young adults (AYA). Ann. Transl. Med..

[44] Berney D.M., Stoneham S., Arora R., Shamash J., Lockley M. (2020). Ovarian germ cell tumour classification: Views from the testis. Histopathology.

[45] Shaaban A., Rezvani M., Elsayes K.M., Baskin H., Mourad A., Foster B.R., Jarboe E.A., Menias C.O. (2014). Ovarian Malignant Germ Cell Tumors: Cellular Classification and Clinical and Imaging Features. RadioGraphics.

[46] Friedman C., Fenster T. (2016). Laparoscopic Treatment of Mixed Malignant Ovarian Germ Cell Tumor in a 16-Year-Old Female Adolescent. J. Pediatr. Adolesc. Gynecol..

[47] Faure-Conter C., Rocourt N., Sudour-Bonnange H., Vérité C., Martelli H., Patte C., Frappaz D., Orbach D. (2013). Les tumeurs germinales de l’enfant. Bull. Cancer.

[48] Luczak J., Bagłaj M. (2018). Ovarian teratoma in children: A plea for collaborative clinical study. J. Ovarian Res..

[49] Kaur B. (2020). Pathology of malignant ovarian germ cell tumours. Diagn. Histopathol..

[50] Boer H., Proost J.H., Nuver J., Bunskoek S., Gietema J.Q., Guebels B.M., Altena R., Zwart N., Oosting S.F., Vonk J.M. (2015). Long-term exposure to circulating platinum is associated with late effects of treatment in testicular cancer survivors. Ann. Oncol..

[51] Blok J.M., Groot H.J., Huele E.H., De Wit R., Horenblas S., Nuver J., Groenewegen G., Bosch J.R., Witjes J.A., Tromp J.M. (2021). Dose-Dependent Effect of Platinum-Based Chemotherapy on the Risk of Metachronous Contralateral Testicular Cancer. J. Clin. Oncol..

[52] Chang Y.-H., Liu H.-W., Chu T.-Y., Wen Y.-T., Tsai R.-K., Ding D.-C. (2017). Cisplatin-Impaired Adipogenic Differentiation of Adipose Mesenchymal Stem Cells. Cell Transplant..

[53] Yao W., Hu Q., Ma Y., Xiong W., Wu T., Cao J., Wu D. (2015). Human adipose-derived mesenchymal stem cells repair cisplatin-induced acute kidney injury through antiapoptotic pathways. Exp. Ther. Med..

[54] Meligy F.Y., Elgheed A.T.A., Alghareeb S.M. (2019). Therapeutic effect of adipose-derived mesenchymal stem cells on Cisplatin induced testicular damage in adult male albino rat. Ultrastruct. Pathol..

[55] Spaeth E., Klopp A., Dembinski J., Andreeff M., Marini F. (2008). Inflammation and tumor microenvironments: Defining the migratory itinerary of mesenchymal stem cells. Gene Ther..

[56] Floyd J., Mirza I., Sachs B., Perry M.C. (2006). Hepatotoxicity of Chemotherapy. Semin. Oncol..

[57] Scuderi M.G., Magro G.G., Di Cataldo A., Pesce A., Scalora L., Vecchio G.M., Portale R., Di Benedetto V., Puleo S. (2013). Evaluation of Neoadjuvant Chemotherapy Effects on Liver Parenchyma in Resected Pediatric Malignancies. Pediatr. Hematol. Oncol..

[58] Kiani A.K., Mor M., Bernini A., Fulcheri E., Michelini S., Herbst K.L., Buffelli F., Belgrado J.P., Kaftalli J., Stuppia L. (2021). Steroid-converting enzymes in human adipose tissues and fat deposition with a focus on AKR1C enzymes. Eur. Rev. Med. Pharmacol. Sci..

[59] Nobili V., Alisi A., Valenti L., Miele L., Feldstein A.E., Alkhouri N. (2019). NAFLD in children: New genes, new diagnostic modalities and new drugs. Nat. Rev. Gastroenterol. Hepatol..

[60] Mattsson C., Olsson T. (2007). Estrogens and Glucocorticoid Hormones in Adipose Tissue Metabolism. Curr. Med. Chem..

[61] Pedersen S.B., Kristensen K., Hermann P.A., Katzenellenbogen J.A., Richelsen B. (2004). Estrogen Controls Lipolysis by Up-Regulating α2A-Adrenergic Receptors Directly in Human Adipose Tissue through the Estrogen Receptor α. Implications for the Female Fat Distribution. J. Clin. Endocrinol. Metab..

[62] DiSaia P.J., Saltz A., Kagan A.R., Morrow C.P. (1977). Chemotherapeutic retro-conversion of immature teratoma of the ovary. Obstet. Gynecol..

[63] Logothetis C.J., Samuels M.L., Trindade A., Johnson D.E. (1982). The growing teratoma syndrome. Cancer.

[64] Amsalem H. (2004). Growing teratoma syndrome vs. chemotherapeutic retroconversion Case report and review of the literature. Gynecol. Oncol..

[65] Kurata A., Takanashi M., Ohno S.-I., Fujita K., Kuroda M. (2021). Cisplatin induces differentiation in teratomas derived from pluripotent stem cells. Regen. Ther..

